# Pre-operative factors correlated with arthroscopic reparability of large-to-massive rotator cuff tears

**DOI:** 10.1186/s12891-019-2485-4

**Published:** 2019-03-18

**Authors:** Vanasiri Kuptniratsaikul, Thongchai Laohathaimongkol, Vantawat Umprai, Chuenrutai Yeekian, Niti Prasathaporn

**Affiliations:** Department of Orthopaedics, Queen Savang Vadhana Memorial Hospital, Chonburi, Thailand

**Keywords:** Rotator cuff tear, Reparability, Prognostic factors

## Abstract

**Background:**

The purpose of this study is to determine the pre-operative factors that are associated with reparability of the large-sized and massive rotator cuff tears.

**Methods:**

Sixty-six patients were included in this prognostic study. Demographic data, radiographic and MRI parameters were collected. Arthroscopic rotator cuff repair was performed for all included patient. Complete rotator cuff repair was achieved when the tendon covered up at least 50% of the anatomical footprint. The receiver operating characteristic (ROC) curve was analysed to define the cut-off level of each significant factor.

**Results:**

Eleven large-sized rotator cuff tears and fifty-five massive rotator cuff tears were defined from MRI. Fifty-four patients were in the complete repair group, and twelve patients were in the partial repair group. The mean duration between MRI and surgery of 5.5 weeks. Reparability was correlated with age, mediolateral (ML) and anteroposterior (AP) tear size, rotator cuff arthropathy, superior migration of humeral head, fatty infiltration and atrophy of the supraspinatus muscle, and fatty infiltration of infraspinatus muscle (*p* < 0.05). The ROC curve defined a cut-off level of each predicting factor which included age of ≥65 years, mediolateral tear size of ≥36 mm, anteroposterior tear size of ≥22 mm, Hamada’s rotator cuff arthropathy of ≥class2, acromiohumeral interval of ≥6 mm, ≥stage3 supraspinatus fatty infiltration, the presence of supraspinatus muscle atrophy, and ≥ stage1 infraspinatus fatty infiltration. In multivariated regression analysis, age, acromiohumeral interval, and anteroposterior tear size were statistically associated with the reparability. The intra- and inter-observer reliabilities were moderate to excellent.

**Conclusion:**

Age, ML tear size, AP tear size, rotator cuff arthropathy, superior migration of humeral head, fatty infiltration of supraspinatus and infraspinatus muscles and supraspinatus muscle atrophy all correlate with reparability of large to massive rotator cuff tear.

## Background

Rotator cuff tear is a common cause of shoulder pain in the general population [1, 2]. There are various surgical approaches available ranging from rotator cuff repair to other salvage procedures such as reverse total shoulder arthroplasty. Arthroscopic rotator cuff repair is the most common procedure for treating large and massive rotator cuff tear as it generally gives satisfactory results in most patients. However, 30% of the cuff tears fail to cover 50% of their own footprint after the surgery [3]. We consider these tears as partial repaired rotator cuff tears which may lead to chronic shoulder pain or functional deficit. There are many treatment options available for symptomatic partial repaired rotator cuff tears such as arthroscopic debridement, arthroscopic biceps augmentation, arthroscopic subacromial spacer, pectoralis or latissimus dorsi tendon transfer, and reversed total shoulder replacement.

Other studies have found that post-operative clinical outcomes are associated with age, operative time, and structural factors, including tear size, anteroposterior and mediolateral tear lengths, tear thickness, proximal migration of humeral head, muscle atrophy, fatty infiltration, and reparability [4–7]. Although a partial repair improves the functional result postoperatively, a complete repair, on the other hand, shows a statistically significant superior functional improvement [8]. Other studies found that tear size, rotator cuff muscle atrophy and rotator cuff muscle fatty infiltration might be associated with reparability of large to massive rotator cuff tear [9–12]. These factors are crucial for creating the guidelines of managing large-sized and massive rotator cuff tear. The purpose of our study is to explore pre-operative clinical, radiographic and magnetic resonance imaging (MRI) parameters, which correlated with arthroscopic reparability of large-to-massive rotator cuff tear.

## Methods

We conducted a retrospective study on prospectively collected data from January 2013 to April 2017. All patients with large-sized or massive rotator cuff tears, which were determined by pre-operative MRI, were included in this study. Our institute classified the rotator cuff tears with modified Millstein and Snyder classification [13, 14]. A large-sized rotator cuff tear was defined as a complete supraspinatus tendon tear. Massive rotator cuff tear was defined as at least two tendons involvement. The tear size was confirmed under arthroscopic examination. Patients with rotator cuff re-rupture or with contraindications for surgery were excluded. Standard plain radiographs and MRI were performed in all patients.

Baseline characteristics including age, gender, duration of symptoms, and traumatic event were collected. Furthermore, radiographic parameters from plain X-ray and MRI including rotator cuff arthropathy, proximal migration of humerus, rotator cuff tear size, fatty infiltration, and muscle atrophy were gathered preoperatively. All parameters were collected twice by each of the two independent observers, in order to compare intra- and inter-observer reliability. However, we used the data from experienced orthopaedist only to analyze for the prognostic factors.

The arthroscopic surgery was performed by three experienced surgeons after the patient was sedated with general anesthesia and set in the beach chair position. In cases of adhesive capsulitis, the capsular release and manipulation was done in the same setting. Acromioplasty was performed if an acromial spur was defined. After the tear size was measured with a probe to confirm the size measured from the MRI, the surgeon released any adhesion surrounding the tendon. The tendon then was mobilized in order to cover its footprint as much as possible. If necessary, the interval sliding technique was also performed. The transosseous-equivalent double-row technique was chosen to be the first line of treatment. If the double-row technique were not feasible, a single-row technique or partial functional repair technique was chosen instead.

**Fig. 1:**
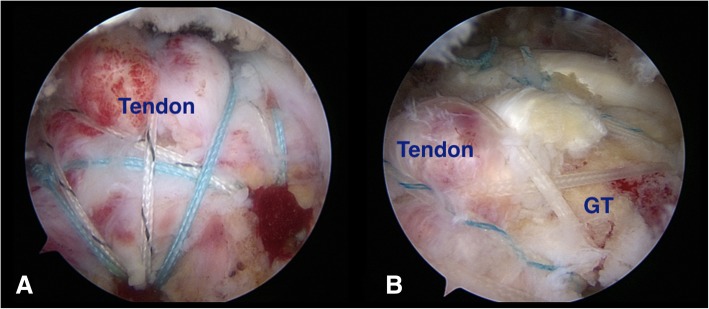
**a** After arthroscopic rotator cuff repair, the tendon covered more than 50% of the anatomical footprint in completely repaired group. **b** In an partially repaired group, the tendon covered less than 50% of the anatomical footprint. (GT = greater tuberosity)

Complete rotator cuff repair was defined as the tendon covering up at least 50% of the anatomical footprint, while partial repair was defined as the tendon covering up less than 50% of the original footprint (Fig. 1) [12, 15]. All patients were underwent passive motion exercises on the first post-operative days. An arm sling was applied for 6 weeks. A progressive active-assisted passive motion exercises were commenced at the third to fourth week for muscle strengthening. This research was approved by Research and Ethic Committee from Queen Savang Vadhana memorial hospital.

### Radiographic parameters

Pre-operative plain radiograph focused on three parameter measurements to evaluate rotator cuff arthropathy which include osteoarthritic change, acromiohumeral distance, and inferior glenohumeral distance. Further pre-operative MRI evaluation focused on mediolateral (ML) tear size, anteroposterior (AP) tear size, tendon retraction, fatty infiltration and muscle atrophy. Radiographic parameters were evaluated by one experienced orthopedist and one senior orthopedic resident. A 1.5-T MRI (Siemens Healthcare, Germany) was used in this study. All measurements were made on a PACS workstation using Agfa IMPAX 6 (Waterloo, Canada) software technology—this software uses data within the DICOM (NEMA, VA, USA) header on all MRI (whether from our institution or from outside institutions) to allow referenced measurements to be made.

### Rotator cuff Arthropathy

The rotator cuff arthropathy was graded by Hamada’s classification [5], which showed good inter- and intra-observer reliability from a previous study [16]. The studied population were classified into 5 grades: grade 1, the AHI is ≥6 mm; grade 2, the AHI is ≤5 mm; grade 3, AHI is ≤5 mm with acetabulization of the acromion; grade 4, glenohumeral narrowing; and grade 5, humeral head collapse [7].

Superior Migration of the Humeral Head; Acromion-Humeral Interval (AHI) & Inferior Glenohumeral Distance (IGHD).

**Fig. 2:**
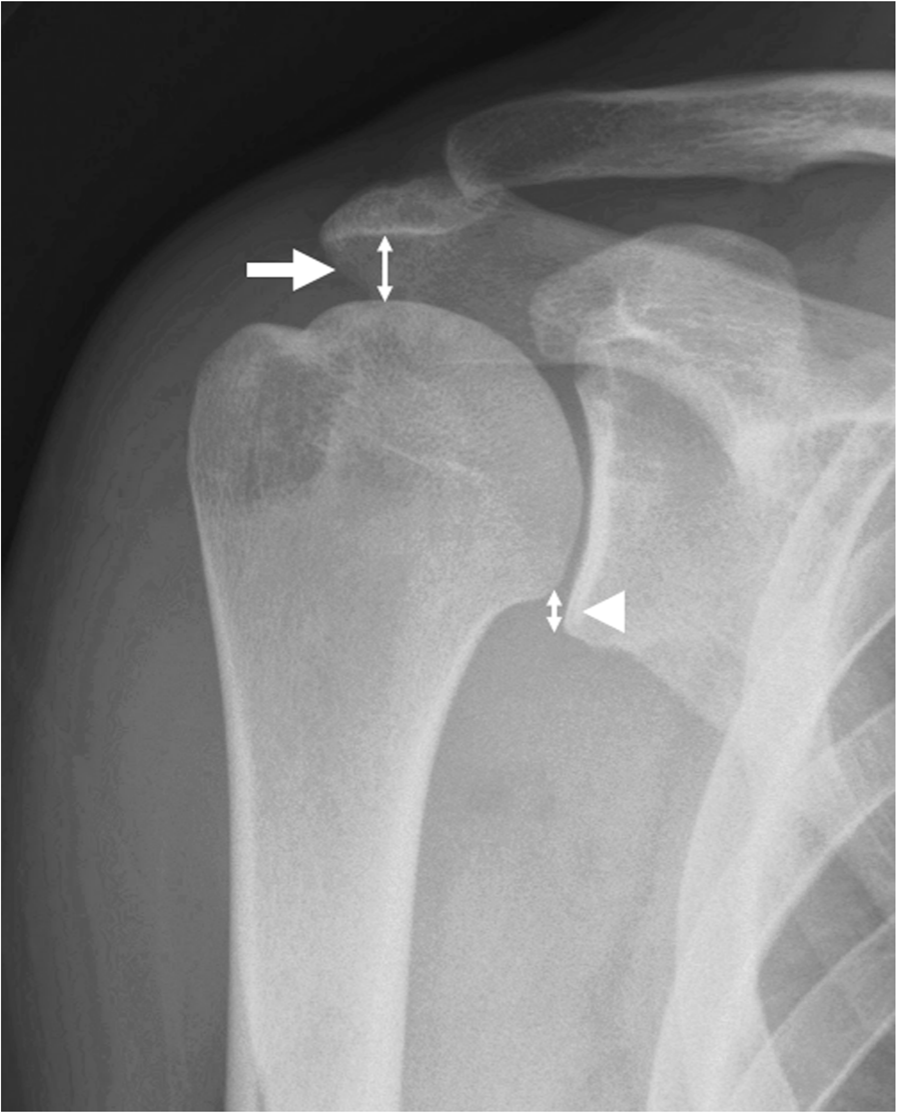
The superior migration of humeral head was defined with two methods of measurement. Acromioclavicular interval (arrow) was measured between the inferior border of acromion and the superior aspect of humeral head. Inferior glenohumeal distance (arrow head) was measured between the inferior glenoid margin and the inferior humeral head margin at just the intersection of humeral head and the humeral neck

Acromion-humeral interval (AHI) was one of the parameter to evaluate superior migration of the humeral head (SMHH). It was measured on an anteroposterior view of the plain radiograph, as the distance between inferior border of acromion and superior aspect of the humeral head. Inferior glenohumeral distance (IGHD) was another parameter to evaluate SMHH measuring the distance between inferior glenoid margin and the inferior humeral head margin at just the intersection of the humeral head and the humeral neck [12] (Fig. 2).

### Tear size

**Fig. 3:**
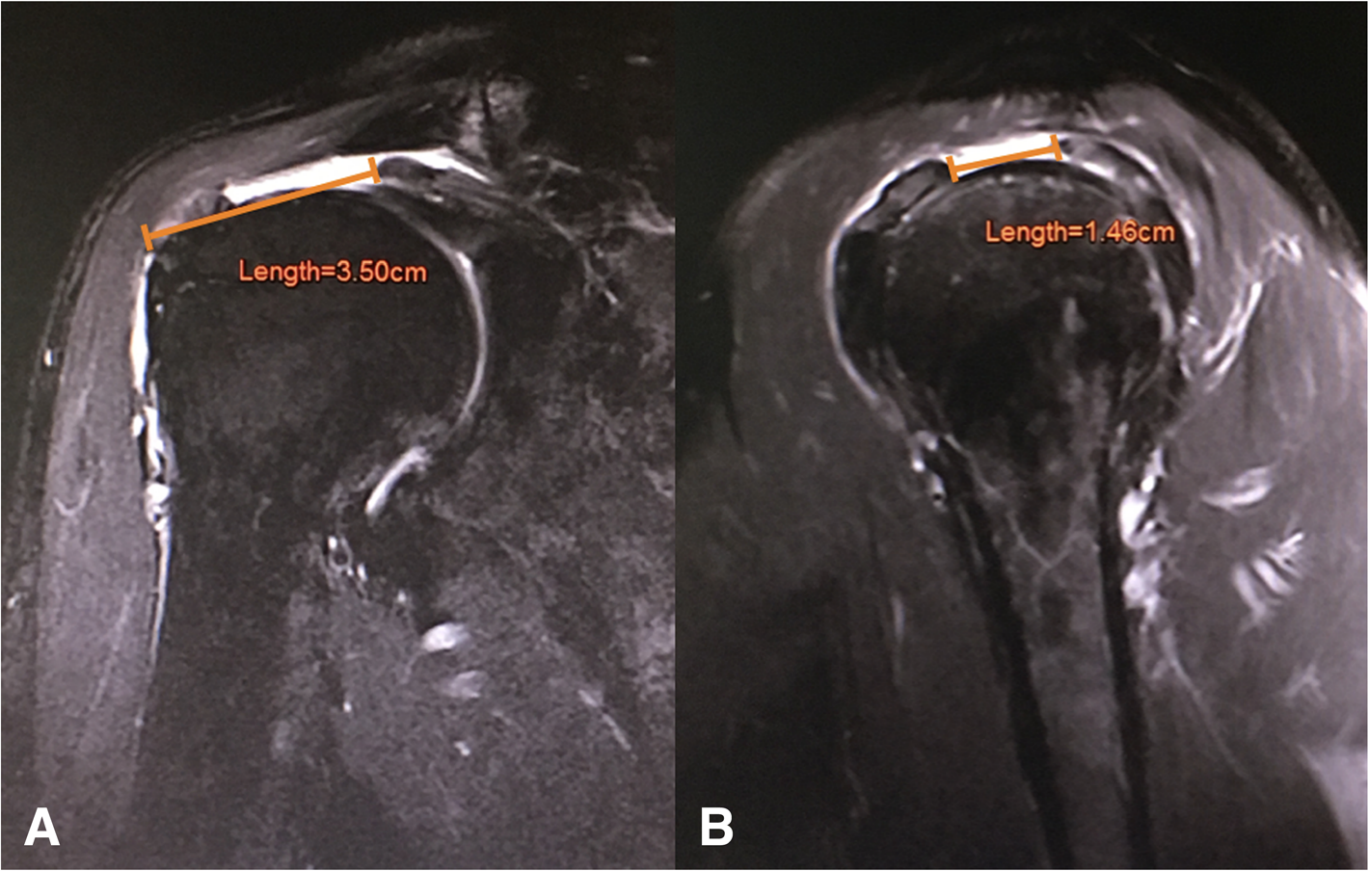
**a** From magnetic resonance imaging, mediolateral tear size was measured between the tendon edge and the lateral cortex of greater tuberosity in the coronal oblique cut, which the tendon most retracted medially. **b** Anteroposterior tear size was measured between the anterior tendon edge to the posterior tendon edge in the sagittal oblique cut, which showed the longest distance

Tear size of supraspinatus tendon was collected from T2-weighted MRI in both coronal oblique and sagittal oblique views which gave mediolateral (ML) tear size and anteroposterior (AP) tear size respectively. ML tear size was a straight distance from the tendon edge to the lateral cortex of the greater tuberosity in the coronal oblique cut, which the tendon most retracted medially. AP tear size was a longest straight distance from the anterior tendon edge to the posterior tendon edge in the sagittal oblique cut [17]. The frayed tissues at the tip of tendon edge were not included in any measurement (Fig. 3).

### Tendon retraction

From the coronal oblique view of T2-weight MRI, we use the slice which showed the most medial tendon retraction. The tendon retraction was classified using the classification described by Patte [18]. In stage 1, the tendon stump was close to the bony insertion. In stage 2, the tendon stump was retracted and lied at the level of humeral head between the footprint and glenoid. In stage 3, the tendon stump was seen at the level of the glenoid or beyond.

### Fatty infiltration

Using the sagittal oblique view of T1-weight MRI, fatty infiltration in all rotator cuff muscles were classified by the modified Goutallier staging system described by Fuch et al. [19]. Based on the lateral image on which the scapular spine was in contact with scapular body (Y-shaped view), the patients were classified into 5 grades: grade 0, normal muscle without fatty streak; grade 1, some fatty streaks in the muscle; grade 2, fatty infiltration is present, but more muscle than fat; grade 3, equal amount of fat and muscle; and grade 4, more fat than muscle.

### Muscle atrophy

**Fig. 4:**
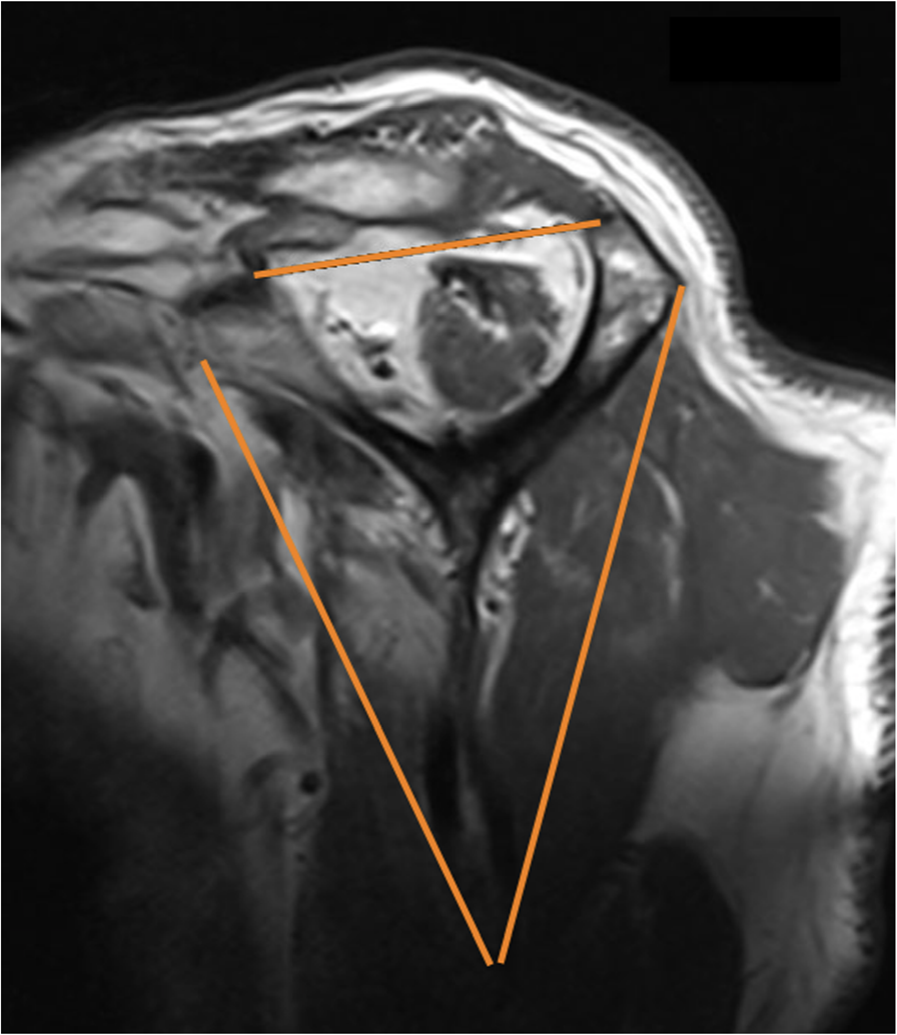
From the sagittal oblique view of T1-weighted magnetic resonance imaging, the muscle atrophy were evaluated in the most lateral image on which the scapular spine was in contact with scapular body (Y - shaped view). The Zanetti’s tangent line extended from superior aspect of the coracoid to the superior border of the scapular spine, then the other two lines were extended from each scapular process to tip of scapular body

We used the sagittal oblique view of T1-weighted MRI to evaluate the rotator cuff muscle atrophy by selecting the most lateral image on which the scapular spine was in contact with scapular body (Y-shaped view). First, the Zanetti’s tangent line extended from the superior aspect of the coracoid to the superior border of the scapular spine and the other two lines were extended from each scapular process to the tip of scapular body (Fig. 4). These lines divided muscle atrophy into 4 groups: none or no muscle atrophy, the muscle crossing over the tangent line; mild, border of the muscle touching the tangent line; moderate, a concave curve of the muscle beside the tangent line; and severe, the muscle bundle atrophy nearly touching the scapular spine and scapular body [20].

### Statistical analysis

As the data were not normally distributed, we selected the Chi-square test to compare proportions of categorical variables between complete and partial repair group. These variables included gender, traumatic event and tendon involvement. A Mann-Whitney U test was used to compare between continuous data variables in the complete and partial repair groups. These include age, duration of symptom, AHI and IGHD, ML and AP tear size, grading of fatty infiltration and muscle atrophy. Pearson-correlation analysis and Spearman rank-order correlation took a role in determining association between categorical and continuous variables, respectively. These variables, which determined the independent factors affecting reparability, were analyzed in a stepwise multivariate logistic regression analysis. We created the receiver operating characteristic (ROC) curve of significant predicting factors which correlated with reparability of rotator cuff tendons. The cut-off levels of each factor were defined from the ROC curve. The likelihood ratios of each factor were then calculated to weight the importance to rotator cuff tendon reparability.

Intra-observer and inter-observer reliabilities were evaluated with the Kappa analysis and intraclass correlation coefficient for categorical and continuous data respectively.

Statistical significant was set at *p*-value < 0.05. We use the IBM SPSS statistics software version 20 for all statistical analysis.

## Results

Sixty-six patients (31 women and 35 men, mean age 60 years, range 25–84 years) were included in the analysis. Eleven large-sized rotator cuff tears (16.67%) and fifty-five massive rotator cuff tears (83.33%) were defined from MRI and all confirmed under arthroscopic examination. The mean duration between MRI and surgery was 5.5 weeks. Overall, 54 and 12 patients underwent complete and partial repair, respectively. Demographic data comparing between each group with respect to age, gender, traumatic event, duration of symptom, duration between MRI to surgery, rotator cuff arthropathy, AHI and IGHD, ML and AP tear size, grading of fatty infiltration, and muscle atrophy as showed in Table 1. There are nine parameters; age, ML tear size, AP tear size, rotator cuff arthropathy, AHI, IGHD, fatty infiltration and muscle atrophy of the supraspinatus muscle, and only fatty infiltration of the infraspinatus muscle which show a significant correlation with partially repaired cases.

**Table 1:** Descriptive data of 66 patients (Complete Repair vs Partial Repair group)

Predictive factors	Complete Repair (*n* = 54)	Partial Repair (*n* = 12)	*p*-value
Age	59.31(range 25–84)	67.75(range 47–81)	.015
Male	27 (50%)	8 (66.67%)	0.295
Duration of onset (weeks)	22.24(range 3–98)	27.54(range 5–104)	0.634
Traumatic event	31 (57.41%)	5 (41.67%)	0.322
Rotator cuff arthropathy			
• Grade 0	15 (27.78%)	0 (0%)	0.004
• Grade 1	33 (61.11%)	5 (41.67%)	
• Grade 2	3 (5.56%)	5 (41.67%)	
• Grade 3	2 (3.70%)	1 (8.33%)	
• Grade 4	1 (1.85%)	1 (8.33%)	
• Grade 5	0 (0%)	0 (0%)	
AHI	7.92 (range 0–15.36)	5.14 (range 1.30–10.21)	0.003
IGHD	3.85 (range (−4.3)-14.36)	6.62 (range (−3.35)-10.85)	0.036
Mediolateral tear size	28.48 (range11.74–48.3)	39.75 (range28–48.27)	.001
Anteroposterior tear size	19.94 (range0–44.56)	30.76 (range10–47.3)	.002
Fatty infiltration (Goutallier staging)			
• Supraspinatus muscle			
ο Stage 0	33 (61.11%)	1 (8.33%)	<.001
ο Stage 1	10 (18.52%)	3 (25%)	
ο Stage 2	5 (9.26%)	2 (16.67%)	
ο Stage 3	3 (5.56%)	5 (41.67%)	
ο Stage 4	3 (5.56%)	1 (8.33%)	
• Infraspinatus muscle			
ο Stage 0	35 (64.81%)	3 (25%)	.031
ο Stage 1	5 (9.26%)	3 (25%)	
ο Stage 2	5 (9.26%)	2 (16.67%)	
ο Stage 3	4 (7.41%)	3 (25%)	
ο Stage 4	5 (9.26%)	1 (8.33%)	
• Subscapularis muscle			
ο Stage 0	36 (66.67%)	5 (41.67%)	.199
ο Stage 1	9 (16.67%)	4 (33.33%)	
ο Stage 2	3 (5.56%)	2 (16.67%)	
ο Stage 3	2 (3.70%)	1 (8.33%)	
ο Stage 4	4 (7.41%)	0 (0%)	
• Teres minor muscle			
ο Stage 0	40 (74.07%)	8 (66.67%)	.701
ο Stage 1	9 (16.67%)	3 (25%)	
ο Stage 2	3 (5.56%)	1 (8.33%)	
ο Stage 3	0 (0%)	0 (0%)	
ο Stage 4	2 (3.70%)	0 (0%)	
Muscle atrophy			
• Supraspinatus muscle			
ο None	33 (61.11%)	0 (0%)	.001
ο Mild	11 (20.37%)	7 (58.33%)	
ο Moderate	7 (12.96%)	5 (41.67%)	
ο Severe	3 (5.56%)	0 (0%)	
• Infraspinatus muscle			
ο None	44 (81.48%)	7 (58.33%)	.163
ο Mild	3 (5.56%)	4 (33.33%)	
ο Moderate	2 (3.73%)	0 (0%)	
ο Severe	5 (9.26%)	1 (8.33%)	
• Subscapularis muscle			
ο None	36 (87.72%)	11 (91.67%)	.639
ο Mild	3 (6.98%)	0 (0%)	
ο Moderate	0 (0%)	1 (8.33%)	
ο Severe	4 (9.30%)	0 (0%)	
• Teres minor muscle			
ο None	47 (87.04%)	12 (100%)	.502
ο Mild	3 (5.56%)	0 (0%)	
ο Moderate	0 (0%)	0 (0%)	
ο Severe	4 (7.41%)	0 (0%)	

The mean age was 59.3 years in the complete repair group and 67.7 years in the partial repair group. This revealed a significant age difference in the partial repair group(*p* = .015). Both ML and AP tear size also showed a correlation with partial repair with *p*-value of .001 and .002 respectively. Rotator cuff arthropathy, which was graded by the Hamada classification, AHI and IGHD were all correspondingly related to partial repair (*p* = .004, *p* = .003, *p* = .036) Fatty infiltration and muscle atrophy of supraspinatus muscle was significantly associated with partial repair(*p* < .001, *p* = .001). Fatty infiltration of infraspinatus was correlated with partial repair(*p* = .031).

The ROC curves were further analyzed on these nine significant predicting factors, age, ML tear size, AP tear size, rotator cuff arthropathy, AHI, IGHD, fatty infiltration, and muscle atrophy of the supraspinatus muscle, and fatty infiltration of the infraspinatus muscle to search for each proper cut-off point that gave optimal sensitivity, specificity and likelihood ratio. The results were as follows; age of 65 years old, 22 mm of AP tear size, 36 mm of ML tear size, class 2 of Hamada’s rotator cuff arthropathy, AHI of 6 mm, IGHD of 5 mm, stage 3 of fatty infiltration of supraspinatus muscle and stage 1 of fatty infiltration of infraspinatus muscle, and mild degree of supraspinatus muscle atrophy gave the optimal sensitivity and specificity (Table 2). The post-hoc analysis was performed to define the power of each significant predicting factor. The power of each factor ranges from 70.2 to 98.2%.

**Table 2:** Results from Receiver Operating Characteristic curve analysis of predicting factors of arthroscopic reparability for large-sized and massive rotator cuff tears

Factor	Cut point	Sensitivity (SD)	Specificity (SD)	Likelihood ratio	*P*-value
Age	≥ 65 years old	0.83 (0.04)	0.70 (0.03)	2.81	0.015
ML tear size	≥ 36 mm	0.91 (0.02)	0.74 (0.03)	3.51	0.001
AP tear size	≥ 22 mm	0.75 (0.05)	0.70 (0.03)	2.65	0.002
Rotator cuff arthropathy	≥ stage 2	0.58 (0.07)	0.89 (0.01)	5.25	0.004
AHI	≤ 6 mm	0.64 (0.07)	0.78 (0.02)	2.86	0.003
IGHD	≥ 5 mm	0.73 (0.06)	0.60 (0.03)	1.79	0.036
Supraspinatus fatty infiltration	≥ stage 3	0.50 (0.07)	0.89 (0.01)	4.50	< 0.001
Infraspinatus fatty infiltration	≥ stage 1	0.75 (0.05)	0.64 (0.03)	2.79	0.031
Supraspinatus muscle atrophy	mild to severe	1.00 (N.A.)	0.60 (0.02)	2.52	0.001

We have found there were only three independent factors that significantly affected the reparability after multivariated logistic regression analysis. These were age of 65 years or older (*p* = .004), acromiohumeral index of less than 6 mm. (*p* = .007), and anteromedial tear size of more than 22 mm. (*p* = .026) (Table 3).

**Table 3:** Significant factors, in multiple logistic regression models, associated with arthroscopic reparability of large and massive rotator cuff tear

Factor	Coeff (Std. err)	OR	95% CI		*P*-value
			Lower Bound	Upper Bound	
Age ≥ 65 years old	0.333	1.395	0.085	0.429	0.004
AHI ≤ 6 mm	0.297	1.346	0.067	0.417	0.007
AP tear size ≥22 mm	0.243	1.275	0.023	0.359	0.026

The intra-observer reliability ranged from 60 to 100% with average 85.69%. The inter-observer reliability ranged from 43.33 to 100% with an average of 74.5%. Both results were considered as good to excellent.

## Discussion

Arthroscopic surgery is now becoming a more for popular option for rotator cuff repair as it shows good to excellent results. However, large-sized and massive rotator cuff tears remain a challenge situation as there is a high re-rupture rate [11, 21–23]. Previously, Burkhart has described a concept of functional partial repair which reduces pain and improves shoulder function [24–27]. Furthermore, recent studies showed that partial repair provide inferior functional outcomes, shoulder mobility and strength compared to the anatomical repair [8]. Tendon mobility to cover the footprint could only be demonstrated under the operation. Nonetheless, various options of arthroscopic and open surgery were performed for the partially repaired rotator cuff tears ranging from arthroscopic debridement to reversed total shoulder replacement as described in the introduction.

The transosseous-equivalent double-row technique is widely used in arthroscopic rotator cuff repairs as it has been shown in several biomechanics studies to have a higher tensile strength than a conventional single-row technique [28, 29]. The previous meta-analysis studies showed that double-row repair had a more complete healing rate, less re-rupture rate, better range of motion, and improved clinical outcomes, especially in large-sized and massive rotator cuff tears [30–32]. Hence, in this study, we intended to arthroscopically repair rotator cuff tendons with transosseous-equivalent double-row technique to completely cover the anatomical footprint as much as possible. In case the tendon is unable to be fully mobilized, single-row repair or functional partial repair was chosen instead.

Other recent studies have explored the clinical and surgical factors in predicting reparability of large and massive rotator cuff tears. A study by Holtby and Razmjou showed that U-shaped tears, tear size, and tendon quality defined intraoperatively correlated with reparability. On a contrary, there is no pre-operative factors that can predict the reparability of large and massive rotator cuff tears [8]. Dwyer et al. studied the association between pre-operative MRI and reparability of large and massive rotator cuff tears. The results were quite similar with previous studies, demonstrating mediolateral tear size, tendon retraction to the glenoid level, muscle atrophy, advanced fatty infiltration and superior migration of humeral head were associated with partial repairs [15].

Our study included clinical, radiographic and MRI parameters to analyze the correlation with reparability of large-sized and massive rotator cuff tears. The rotator cuff tear is a degenerative disease as Pecora et al. demonstrated that age is associated with clinical outcomes after rotator cuff repair [33]. From our study, age is one of the factors that correlates with rotator cuff reparability. The tendon tissue quality, which differs in various age groups, is also likely an important factor. The mechanism of rotator cuff tear differs among different age groups. Younger patients often presented with acute traumatic tears, while older patients often presented with chronic degenerative tears. It is found that the likelihood ratio of partial repair is 2.81 in patients 65 years or older. In traumatic rotator cuff tears, there is more adhesion and scar in a subacromial space. Although the adhesion limits the tendon mobility and makes a repair more difficult, a traumatic event does not correlate with reparability in this study.

Our study showed that the onset of the symptom is not correlated with AHI, fatty infiltration, muscle atrophy, and rotator cuff arthropathy, all of which are the parameters of chronicity. One explanation might be the recall bias from the patients. Nevertheless, this aspect supports that rotator cuff tears are asymptomatic may be silent for certain period of time before they were clinically detected.

This analysis showed stage 2 or more of Hamada’s classification is related with high risk of partial repair. Our study also found that in stage 2 of Hamada’s classification with less than 5 mm of AHI, the superior migration of humeral head significantly correlated with partial repair, similar to prior studies as well as IGHD that was associated with the reparability [15]. Regardless, our study shows AHI 6 mm or more and IGHD 5 mm or more correlate with partial repair of rotator cuff tears with the likelihood ratio of partial repair of 2.86 and 1.79, respectively.

For MRI parameters, there were previous studies which demonstrated the association between tear sizes and partial repair [34]. Yoo et al. reported the sagittal tear size of equal to or greater than 32 mm and the coronal tear size of equal to or longer than 31 mm correlated with an inability to achieve satisfactory anatomical rotator cuff repair [21]. Sugihara et al. showed that primary rotator cuff repair was usually not feasible when both the length and width of tear are equal to or longer than 40 mm [34]. Our study demonstrated a mediolateral tear size of 36 mm or more and anteroposterior tear size 22 mm or more correlated with partial repair of rotator cuff tears with likelihood ratio of partial repair of 3.51 and 2.65, respectively. Tendon retraction, a parameter that can resemble the tear size, but more simple to use and more reliable. On a contrary, it is not significantly correlated with reparability.

Advanced fatty infiltration, which was described by Goutallier et al., correlates with an inability to achieve complete rotator cuff repair [15, 21]. Our study demonstrates advanced fatty infiltration of supraspinatus muscle (stage 3 or more) and mild degree of infraspinatus muscle (stage 1 or more) correlated with an unsuccessful rotator cuff repair with the the likelihood ratio of partial repair of 4.50 and 2.79, respectively. The classification of muscle atrophy, which Thomazeau et al. described, is widely used [35]. From our study, the muscle atrophy of stage I or greater, where the muscle mass is just at the same level of a tangent line or below, correlated with partial repair with the likelihood ratio of 2.52.

The limitation of this study is a low number in the partial repair group. Comparing to prior studies [8, 15, 34], the rate of partially repaired rotator cuff tear in our study is quite different, despite the fact that we use the same criteria of reparability. There was a broad range of age group, which may impact the tissue quality and probability of traumatic rotator cuff tear. However, the post-hoc analysis showed the powers of each significant predicting factor range from 70.2 to 98.2% despite only 12 patients in partial repair group.

## Conclusions

Reparability of large-to-massive rotator cuff tears correlate with the following factors; age, AP tear size, ML tear size, rotator cuff arthropathy, AHI, IGHD, fatty infiltration of supraspinatus and infraspinatus muscles, and atrophy of supraspinatus muscle.

## Abbreviations

AHI: Acromiohumeral interval
AP: Anteroposterior
IGHD: Inferior glenohumeral distance
LR: Likelihood ratio
ML: Mediolateral
MRI: Magnetic resonance imaging
ROC: receiver operating characteristic
SMHH: Superior migration of humeral head
